# The Chain of Custody in the Era of Modern Forensics: From the Classic Procedures for Gathering Evidence to the New Challenges Related to Digital Data

**DOI:** 10.3390/healthcare11050634

**Published:** 2023-02-21

**Authors:** Tommaso D’Anna, Maria Puntarello, Giovanni Cannella, Giovanni Scalzo, Roberto Buscemi, Stefania Zerbo, Antonina Argo

**Affiliations:** 1 General Hospital, University of Palermo, Via del Vespro 129, 90127 Palermo, Italy; 2 Department of Health Promotion, Maternal and Child Care, Internal Medicine and Medical Specialties, Section of Forensic Medicine, University of Palermo, 90127 Palermo, Italy

**Keywords:** forensics, evidence, chain, custody, forensics, digital data, guidelines, innovative technologies

## Abstract

The purpose of this work is to renew the interest and attention for the chain of custody in forensic medicine, its establishment and maintenance, protecting the integrity and validity of evidence as well as to analyze how over time the establishment of the chain of custody and the collection of evidence has evolved also in function of the advent of technology and the use of electronic devices connected to the network. The analysis of the various aspects of the chain of custody demonstrates how necessary it is for the professional figures involved in the phases of the investigation (especially those who manage the evidence and who have, therefore, designated the assignment) to know the procedures to follow, trace the movement and the handling of objects subjected to seizure, also for the purposes of toxicological and/or histological investigations. The knowledge of interferences or complications helps to reduce errors and safeguard the validity of the evidence, assuring the proceeding judicial authority that the evidence is authentic and that it is, in other words, the same evidence seized at the scene of the crime. Furthermore, the issue is particularly felt today, with the recent need to guarantee the originality of digital data. Following a careful review and analysis of the literature currently available in this regard, it is worth adding that further efforts are needed to formulate internationally validated guidelines, harmonizing the different reference criteria in forensic science and medical areas, given the current absence of good international practices valid in the field and applicable both in the case of physical evidence and in the case of seizure of digital evidence.

## 1. Introduction

Chain of custody is the most important and, at the same time, the most critical process of documenting evidence: in criminal and civil law, the term “chain of custody” refers to the order in which evidence was dealt with during the investigation of a case [1]. When a chain of custody is required, to show the artifact’s authenticity or its unchanged condition, it is necessary to determine where the chain begins and ends. As reported in a work by Houck and colleagues, only breaks in possession occurring within the chain of custody period affect eligibility [2].

It is essential to assure the judicial authority that the evidence is authentic and that it is the same that is seized at the scene of the crime. The fundamental point of correct maintenance of the chain of custody consists in the possibility of access to the original exhibit. Consequently, it is appropriate that each person in charge of its custody is fully aware of the responsibility of keeping the evidence intact and of tracing, through suitable documentation, each passage or conservation [3], which ensures the integrity of the chain of custody.

## 2. Material and Method

**Figure 1:**
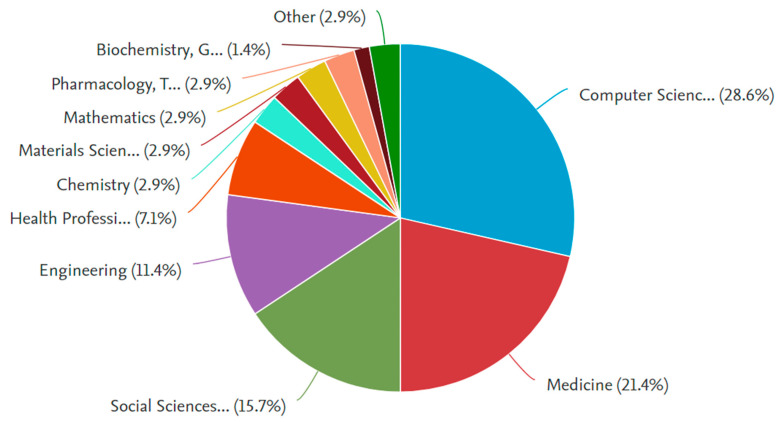
Documents’ distribution by thematic area, concerning CC and F.

In this narrative review, we look for the themes of chain of custody (CC) and forensics (F), considering aspects of important and innovative role in the near future. Three main databases and indexed bibliographic searches were studied. The PubMed database showed 71 articles in English, accessible in an extended version; 45 were considered as subjects of interest, in the years 1990–2022 25 articles corresponding to topics CC and F were considered as review articles [4,5,6,7,8,9,10,11,12,13,14,15,16,17,18,19,20,21,22,23,24,25]. No clinical studies were found. We considered 2 systematic reviews [4] addressing cases of violence and abuse and population-level and individual-level DNA databases, respectively, for forensic identification and chain of custody certification [5]. In the Scopus database, 105 articles matching this field were found within keywords, covering the period 2004–2022. By limiting the area of interest to computer science, and medicine and the healthcare profession, 25 articles and 5 reviews were accessible and helpful for this topic. Worthy of consideration, subjects of interest included the traditional subjects of forensic pathology, toxicology, and forensic entomology; in the last decade, research topics have focused on the blockchain system and on innovative technologies applied to the forensic standard [13,14,15,16,17,18,19,20,21,22,23,24,25,26,27,28,29,30,31,32,33,34,35]. The number of 21 documents (duplicates and not accessible) were not considered useful for the research. In the area of interest in computer science and medicine and healthcare, 25 articles and 5 reviews have been accessible and helpful for this review. On the MDPI database, 6 articles were found available from 1996 to 2023 by searching for themes CC and F; 3 accessible articles were found useful and used for the study of the topic. The distribution of documents by subject area of CC and F is shown in Figure 1.

## 3. Discussion

### 3.1. The Importance of the Chain of Custody

The chain of custody demonstrates the integrity of an item of evidence [34,35,36]. A paper trail should be maintained so that the individuals who supervised the preservation of evidence at any given time can be recognized and summoned to testify at trial if the need arises. As highlighted in a study by Jaffe and colleagues, a chain of custody control of evidence must be established whenever an object is presented as evidence [37,38]. Otherwise, the evidence may be considered inadmissible, casting serious doubts on its authenticity/integrity (also considering the possibility of adulteration and contamination of the sample) and on the tests carried out on them such as, for example, toxicological or histological tests [39,40], in usual or unusual, or “alternative”, matrices [41,42]. Proper chain of custody has been a crucial factor in high-profile cases, such as the 1994 murder trial of former pro football star O.J. Simpson [1,43].

The chain of custody must contain and document every transmission of the object from person to person since the seizure. The goal is to establish that the evidence is related to the alleged crime, was collected at the scene, and was in its original/unaltered condition rather than having been tampered with or otherwise polluted [1]. To convict a defendant of a crime, the evidence against him or her must have been meticulously handled to avoid tampering or contamination.

The traceability of the registration of the control, of the transfer, and of the analysis of the samples indicates the transparency of the procedure [44]. Maintaining the chain of custody is critical in forensic practice. Indeed, chain of custody documentation should be complete with information regarding the circumstances of the collection of evidence, the conditions of custody during the handling and/or retention of evidence, and how evidence is handed over to subsequent custodians each time that a transfer occurs (together with the signs of the people involved in the respective phase).

### 3.2. Chain of Custody Documentation

Chain of custody documentation has three main purposes: to ask the testing laboratory pertinent questions about the tests, to maintain a chain of custody record, and to document that the sample/test was handled only by authorized personnel and was not accessible for tampering prior to the analyses [3,44,45]. The problem of maintaining the chain of custody also has an important resonance in the evidence maintained by the Intelligence, so much so that an attempt has been made to set up a system called Disciple—LTA, where LTA means “learner, tutor and assistant”. This system allows analysts to perform credibility assessments on the original sources, as well as to evaluate possible uncertainties that arise in the maintenance of the chain of custody [46].

The Investigator or person responsible for collecting evidence must complete the sample container/bag labels and chain of custody forms to enable sample traceability. Each sample container label must bear a unique identification number and other pertinent information such as location, date and time of collection, name and signature of the person who collected the sample, and signature(s) of the witness(es). It is essential that evidence is properly packaged to avoid damage in transit and should preferably be sealed in tamper-evident bags or with tamper-evident tape.

- During the investigation, the different chain of custody officers should follow several steps [2]:
- document the location of the scene and the time of arrival of the investigator and coroner;
- determine evidence repository(s), determine which laboratory(ies) is/are responsible for collecting specific types of evidence, and determine evidence collection priority for fragile evidence;
- identify, document, protect and preserve evidence with appropriate containers, labels and preservatives;
- document evidence collection by recording its on-site location, time of collection, time and place of disposal, and by whom;
- develop personnel rosters, witness lists, and documentation of personnel arrival and departure times.

A separate chain of custody form must accompany the different sets of evidence. The chain of custody form—in order not to be considered invalid for the purpose of demonstrating the correct maintenance of the chain of custody—must contain at least the following information [3,47]:

- unique identifier;
- name and signature of the person building the sample;
- address and telephone number;
- details of each sample;
- type of analysis required.

However, there are no unique forms that document the chain of custody, and lists and registers with different characteristics can also be used [47].

According to an authoritative study by Schum and colleagues, with the development of technology we have worked and will continue to work to increasingly adapt the paper documentation of the chain of custody to digitization, mainly using secure and traceable IT systems [46]. This modus operandi is already in use in the United States through the establishment of the HORIZON LIMS (laboratory information management system), which provides for a fully automated control through the use of containers with a unique code then registered on the system which controls and certifies their chain of custody through the use of electronic signatures that can be affixed by staff with a unique ID and an encrypted password. To date, this is the most reliable and complete LIMS traceability method [48].

### 3.3. Evidence Custody

Whenever evidence is used for the purposes of the investigation, the signature, date, and time must be entered for the chain of custody form. A sample is in custody if it is actually in the physical possession of the authorized custodian in a secure location without access to unauthorized personnel or exposed to any possibility of tampering.

During the trial, if the defense attorney raises concerns about the chain of evidence, the records must show that the chain of custody was never broken. If the inconsistencies persist and the prosecution is unable to prove the chain, it is deemed to have been broken and the defense attorney can seek the annulment of the resulting evidence in court. The same applies to the elements under examination that require toxicological analyses to be performed on them (blood or urine), in these cases the laboratories we rely on will have to comply with certain quality and safety standards that allow the validity of the results deriving from the studies carried out [49,50], as well as demonstrating, if necessary, the non-interruption of the chain of custody.

### 3.4. Other Chain of Custody Usage Scenarios

In addition to crime scene investigations, other areas that find use of chain of custody include [51,52,53]:

- civil litigation;
- doping tests of athletes;
- manage the origin chain, for example, to improve the traceability of food products;
- the supply of medicines;
- seizure of controlled/prohibited substances;
- seizure of money/gold ornaments/other valuables by the customs, tax, or revenue offices;
- in cases of violence and abuse, with adequate training, also of the health professionals involved in the collection of evidence.

### 3.5. Criticality of the Chain of Custody

The study of the literature has made it possible to identify multiple critical issues in the chain of custody relating to the seizure of physical and digital evidence. The first and probably biggest problem is the inadequate packaging of evidence: when it is recovered, it must be protected from tampering. If the forensic expert does not personally package the evidence, due to his greater scientific knowledge, he is responsible for clearly explaining to law enforcement which is the optimal packaging for each type of exhibit. Poorly sealed packages are another potential problem: holes in evidence seals or packaging can lead to loss of evidence or the introduction of contamination. Another potential problem is the loss of recovered items or their disappearance. For this reason, it is important that the figures who intervene on the crime scene draw up a list of the stolen and kept objects.

Another problem could be the pollution of the crime scene by professionals who intervene before the police and investigative experts, such as rescuers whose sole objective is to protect the patient’s health. By intervening, they can alter, make dirty, or contaminate the crime scene, compromising the validity of the evidence [54,55,56,57]. The loss of the chain of custody, in the absence of certification requirements, also invalidates the possibility of using any biological or digital object as a source of evidence, implying possible forms of liability of forensic professionals [9,15].

### 3.6. Computer Display of Chain of Custody

The technological progress that has affected the field of data production and acquisition, culminating in the digitization and storage of information on electronic devices, has had clear repercussions on the methods of transmission and storage of the same. In fact, in the forensic field, the guarantee of the authenticity and integrity of the digital information, contained in text files or in images acquired with digital photographic equipment, is an essential prerequisite for the correct conduct of the investigations, and for the use of these pieces of information in judicial proceedings [58,59].

Therefore, nowadays, since the digital medium is one of the main tools used for the acquisition and production of evidence sources, it is necessary to guarantee the integrity of the chain of custody as an indispensable principle to ensure the correctness and the authenticity of the information contained therein.

If, on the one hand, the contribution of computer technology has contributed to making many procedures more efficient, rapid, and precise, on the other, it has equally easily led to the vulnerability of the information contained in files and digital media. A photograph or a text file produced with digital media is easily modifiable and, therefore, alterable: this creates the need to protect the authenticity of the computer artifact by relying on a chain of custody [60]. In this regard, an interesting starting point for reflection is provided by the study by F.E. Salamh and colleagues which highlighted the possible flaws and deficits of the use of cutting-edge systems in the process of collecting and using data, such as flying drones [61]. It is true that, on the one hand, there has been a simpler and more complete collection of information, such as data on air traffic or the collection of data useful for forensic purposes; on the other hand, however, these systems are easy for hackers to interact with and access via WLAN networks, attacking the systems by altering the files which are then transmitted to the users and which, therefore, do not reflect the original data, making the evidence collected absolutely unusable for forensic purposes, given the interruption of the chain of custody.

In the exercise of his or profession, the forensic scientist is often called upon to perform unrepeatable acts, such as the search for “sources of evidence” during a judicial inspection, or to conduct autopsy investigations; in these circumstances, he is called upon to produce photographic documentation aimed at “crystallizing” the crime scene, documenting the existence of artifacts, and drawing up reports whose authenticity and genuineness of content are essential requisites. Likewise, the correctness of the procedures underlying the conservation and transmission of such information, for which a chain of custody is invoked, constitutes a guarantee of reliability and truthfulness of these finds, essential characteristics for the correct orientation of judicial investigations and for their use corrected in legal proceedings.

In the field of photography, which in forensic medicine plays a central role in the documentation of data and circumstances of forensic interest, a great advantage in guaranteeing data security and quality is given by the use of the RAW image format. The RAW format of an image varies depending on the digital camera used: each manufacturer has its own proprietary RAW format. A RAW image is not yet processed or ready to be printed: it is, in fact, the format that allows you to store more data and information in a photographic shot. Manipulation, understood as tampering, of a RAW image is extremely difficult. The post-production of the RAW image, in fact, determines the conversion of the format and the loss—total or partial—of the EXIF metadata contained in the original file; therefore, a further contribution made by information technology to the implementation of a valid chain of custody of the digital record is the reliable timestamp. Several companies offering digital services provide access to this technology, thanks to which it is possible to stamp a certain date and time on the document, even if it has not been digitally signed. The trusted timestamp ensures certainty and authenticity of date and time for a computer document when it is applied. It consists in the generation, by a time-stamping organism, of a digital signature of the document which can be additional to that of the signatories and, therefore, also affixed to files not previously signed. Reliable timestamping can contribute to the correct maintenance of the IT record chain of custody as it represents documented proof of the existence of a given file at a given instant. The documents to which the timestamp has been affixed are enforceable against third parties in the event of litigation [62]. Digital evidence and the virtual world that holds it are very complex, fragile, and, at the same time, long-lasting. The latest guidelines regarding the establishment of digital evidence and their chain of custody date back to 2007 and were issued by the Association of Chief Police Officers. From 2007 to today, the techniques for collecting sensitive data and digital evidence, useful for forensic purposes, have undoubtedly evolved as well as their archiving tools. It is essential for those who have contact with the collection of evidence, their custody, and their analysis, to have a complete and informed view of all the techniques used today and to carefully study the possible weak points of these processes in order to ensure the protection of the information collected. It is necessary to elevate knowledge and adapt it to modern times [63].

The current state of the art on preserving the authenticity of digital data and which could find a successful use in the chain of custody of digital artifacts is represented by two innovative technologies, the blockchain and the NFT.

Blockchain technology is mainly used in the financial sector—it is well known as the technology behind the creation of the Bitcoin cryptocurrency. Blockchain is a shared, immutable record of transactions. Whenever a transaction is performed, all data related to it are stored as a block in the chain. The timing and sequence of transactions are guaranteed; furthermore, the fact that the blocks in the chain are tightly connected to each other prevents any possibility of data tampering. The application of the blockchain in the medical field would allow a very high level of protection, as the data would be reliable and practically impenetrable; however, the blockchain allows data to be updated by those with authorized access [64].

NFTs (Non-Fungible Tokens) are blockchain-based tokens that represent a unique asset, be it digital or physical, thus providing a certificate of authenticity and non-reproducibility for that asset. The use of NFTs is becoming more and more common, from the art world to the automotive industry. The application of blockchain technology and NFTs in forensics could lead to new tools and instruments to ensure the reliability and integrity of digital evidence to the coroner [65]. From the careful review of the relevant literature, however, it has been noted that every technology used to date in order to prevent the leakage of information or alterations of the same, has weaknesses which are those used by criminals who tamper with the evidence. Although blockchain systems are traced at every step through the identification of the unique ID via password and authorization, i.e., the history of all the activities performed on a certain document, there are some flaws that make the acquisition of evidence unusable for forensic purposes. In this regard, the study by Mohamed Ali and colleagues expresses how a single data protection and sharing system, such as a blockchain, is not enough, but it would be useful to use multiple systems simultaneously such as blockchain and fuzzy hash function that can safeguard the acquired data [14].

## 4. Conclusions

Given the importance of the establishment of the chain of custody and its correct management in the various phases of evidence analysis, it is essential in the future to properly train the personnel involved in the collection, transport, and analysis of samples [66]. For example, in the case of bioterrorism or biocrime, it will be essential to maintain high-quality standards which also include correct and secure management of the chain of custody, as well as the certification of analysts and laboratories that analyze and consult the exhibits [67,68]. The same goes for all healthcare professionals who take care of the woman or child subjected to sexual violence [69,70,71,72]. Supporting the need to ensure high-quality training of personnel handling evidence seized at the crime scene are the results of a study by Sievers and colleagues demonstrating increased quality of chain of custody and retention of biological samples of trained personnel [73]. Proper training is also a must for rescuers who often intervene at the crime scene at a time when evidence collection has not yet been completed [74]. The aim should also be to equip the medical personnel involved with belts with waste containers to avoid contamination of the crime scene [52].

Specialist doctors who may find themselves involved in the seizure of evidence should also be aware of the need to create and maintain a chain of custody of the evidence. An example could be the surgeon who has to extract a bullet from the body of a patient [75,76,77,78,79]. Before using the correct precautions to avoid contaminating the evidence, it is advisable to proceed with a meticulous crystallization of the crime scene and of the evidence through photographic surveys carried out from all angles. Subsequently, the samples are taken and stored according to the characteristics of the test itself, in the presence of eyewitnesses. The envelopes with the unique code must, therefore, be sealed with tamper-evident adhesive tape and everything must be recorded on special forms. From now on, each transmission of evidence will have to be recorded and monitored through paper documentation, validated through the signatures of those responsible for custody until the end of the criminal or civil proceedings (or other term established by law). An overview of international guidelines focusing on CC and F confirmed the interest in harmonizing in the European Union, as demonstrated by the guidelines within countries and legal systems. With the financial support of the Prevention and Fight against Crime Program of the European Union European Commission—Directorate General for Internal Affairs, Guidelines for Forensic Laboratory Management Practices Introduction, Responsibility towards Employers, some aspects of the chain of custody, as forms of good practice; they could be the starting point for a definitive elaboration of safe and accepted practices in the various fields of forensic application [80]. Therefore, the employer relies on the forensic professional manager to develop and maintain an effective CC system [81]. Revised codes of conduct and practice for forensic science providers and criminal justice system professionals are provided by the Department of Justice’s National Forensic Science Commission [82,83]. Finally, some interesting critical issues related to the CC are analyzed by Appuhamy, who underlines above all the difficulty of maintaining the CC in hospital healthcare systems that carry out forensic activities [84]. At the end of the literature review, uniformly encountered critical aspects emerged which provide for the correct collection of evidence, both physical and even more digital, their maintenance and their transmission guaranteeing a constant maintenance of the chain of custody, but it was found the absolute lack of a system that is completely safe and protected from external attacks [85,86,87,88]. Furthermore, at present there is no univocal and international protocol that outlines the structuring of the collection processes. Especially when it comes to digital forensic evidence, a secure and efficient system free from the risk of tampering is lacking. The use of internet connections makes the acquired information more vulnerable, although it is easier to preserve and archive them. In the future, it would be of fundamental importance first of all to set up multidisciplinary teams that envisage the presence of ultra-specialist figures such as computer technicians, engineers, toxicologists, coroners, judicial police personnel dedicated to digital evidence who can analyze a unique collection, storage and transmission system evidence that it is made up of multiple elements of safeguarding the information itself, using multiple technologies simultaneously that make the system inaccessible. An accurate study of the instrumentation and software to be used could allow the highlighting of the weak points of this system, protecting it from possible external attacks, thus ensuring the probative effectiveness of the evidence in court, allowing for a demonstration of the validity of the forensic chain of custody [88]. These are the elements on which, in our opinion, we need to work. Keeping in mind the importance of maintaining the chain of custody for forensic purposes, we propose to carry out trials that have, as a starting point, the critical issues identified in the literature in order to improve their effectiveness, efficacy, and, above all, safety.

## Data Availability

Not applicable.
